# Uncovering Hierarchical Regulation among MYB-bHLH-WD40 Proteins and Manipulating Anthocyanin Pigmentation in Rice

**DOI:** 10.3390/ijms23158203

**Published:** 2022-07-26

**Authors:** Xingming Sun, Zhanying Zhang, Jinjie Li, Hongliang Zhang, Youliang Peng, Zichao Li

**Affiliations:** 1 State Key Laboratory of Agrobiotechnology, College of Agronomy and Biotechnology, China Agricultural University, Beijing 100193, China; sungene@cau.edu.cn (X.S.); zhangzhanying@cau.edu.cn (Z.Z.); lijinjie@cau.edu.cn (J.L.); zhangl@cau.edu.cn (H.Z.); 2 Beijing Key Laboratory of Crop Genetic Improvement, College of Agronomy and Biotechnology, China Agricultural University, Beijing 100193, China; 3 Ministry of Agriculture Key Laboratory of Pest Monitoring and Green Management, College of Plant Protection, China Agricultural University, Beijing 100193, China; pengyl@cau.edu.cn; 4 Sanya Institute of China Agricultural University, Sanya 572025, China

**Keywords:** rice, anthocyanin, MBW complex, hierarchical regulation, molecular design

## Abstract

Anthocyanins accumulate in various organs of rice, and the regulatory genes involved in pigmentation of specific organs, such as pericarp, hull, leaf, apiculus, and stigma have been elucidated. However, the corresponding gene for rice culm pigmentation has not been clarified. The well-known MYB-bHLH-WD40 (MBW) complex plays vital role in regulating the anthocyanin biosynthesis pathway in plants. However, the core members of MBW and the hierarchical regulation between these members are not fully elucidated in rice. Here, by map-based cloning, we identified the culm-specific pigmentation gene *S1* whose alleles are also known for hull/pericarp pigmentation. We also clarified that one WD40 protein encoding gene, *WA1*, is indispensable for anthocyanin biosynthesis in rice. In the cascading regulation among MBW members, *S1* (bHLH) acts as the master gene by activating the expression of *C1* (MYB), and then C1 activates the expression of *WA1* (WD40), which is unique in plant species. This enables MBW members to be coordinated in a common way to efficiently regulate anthocyanin biosynthesis genes. Based on these studies, we explored the minimal gene set required for anthocyanin biosynthesis in rice. These findings will help us design new rice varieties with anthocyanin accumulation in specific organs as needed.

## 1. Introduction

Anthocyanins are a kind of flavonoid that is derived from the phenylpropanoid pathway and occur as the major flower pigments in plants [1]. After being catalyzed by a cascade of enzymes which are encoded by structural genes, many kinds of anthocyanins containing different modifications (such as glycosylation, acetylation) were synthesized [2,3]. These anthocyanins were then transported to the central vacuole through specific transporters [4]. The main anthocyanin compounds are cyanidin, pelargonidin, and delphinidin, which have colors varying from orange-red to violet blue [5]. The varying degrees of color are also affected by vacuolar pH [6]. The structural genes involved in the biosynthesis of anthocyanins are regulated by the MBW complex, which has been widely studied in crops, horticultural and model plants [7].

In rice, anthocyanins can accumulate in both vegetative and reproductive tissues, and the major anthocyanin compounds are cyanidin 3-glucoside and peonidin 3-glucoside [8,9]. The structural genes responsible for anthocyanin biosynthesis have been investigated in the past several decades in rice [10,11,12,13]. Among them, only one dihydroflavonol reductase (DFR) encoding gene has functional variations in the natural germplasm. The causal SNP resulted in premature stop-coding of the protein, which is unable to reduce colorless dihydroflavonols to anthocyanins [14]. It has been clarified that the *C-S-A* gene system regulates rice anthocyanins pigmentation in which *C1* (MYB) acts as a color-producing gene and *S1* (bHLH) determines tissue-specific pigmentation [15]. These two kinds of transcription factors have been well studied in the past. For the cloned MYB genes related to anthocyanin biosynthesis, *C1* is one of the important members and plays an essential role in activating the biosynthesis genes. It has a wide range of loss-of-function variations in *indica* and *japonica*, leading to no anthocyanin accumulation in most cultivated rice [16]. *OsKala3*, also known as *MYB3*, is responsible for anthocyanin biosynthesis in pericarp. A structural variation of tandem repeats in the promoter of *OsKala3,* confers diverse expression of it, and provides a rationale for the black pericarp [17,18]. As another member of the MBW complex for anthocyanin biosynthesis, the bHLH transcript factors determine the tissue/organ specificity of pigmentation. The purple-blackish color in pericarp originated from ectopic expression of the bHLH gene *Kala4* due to a DNA fragment insertion in the promoter region [19]. A novel allele of *Kala4*, *S1*, determines the hull pigmentation in rice [15]. *OsPs* and *OsPa*, both of which are located next to *S1* and encode bHLH transcription factors, are specific for stigma and apiculus pigmentation, respectively [20]. *OsRb* also encodes a bHLH and specifically regulates anthocyanin pigmentation in leaf tissues [21]. In addition to anthocyanin pigmentation in rice, the red color in pericarp owing to the accumulation of proanthocyanidins is determined by *Rc* which also encodes a bHLH [22]. Although bHLH TFs regulating various organs’ pigmentation have been discovered, the candidate gene specifically regulates culm coloration of rice is yet to be identified. Besides, WD40 proteins are important partners for MYB and bHLH and play essential roles in the formation of the MBW complex. In rice, only one gene, *OsTTG1*, which encodes a WD40 protein was found to be responsible for anthocyanin pigmentation in pericarp [23]. The MBW members for anthocyanin biosynthesis have been identified in rice, but the coordination between these regulators in promoting expression of the structural genes for tissue-specific pigmentation is still not elucidated.

In addition to regulating structural genes, the hierarchical regulation among MBW members themselves plays important roles in coordinating these factors in a common way and manipulates the activation of downstream structural genes. In *Arabidopsis*, the ectopic expression of *TT2* (MYB) in roots induces the transcriptional activation of *TT8* (bHLH), but it does not affect the expression level of *TTG1* (WD40) [24]. It also known that the *TT8* mRNA was decreased in siliques of the *ttg1-1* mutant, which suggests that *TTG1* is at least required for normal expression of *TT8* in siliques [25]. Combining these results, it is considered that *TT2* and *TTG1* may act upstream of *TT8*. It is interesting to note that it is still not known how anthocyanin-related MBW members regulate each other in rice.

Through in-depth study of the molecular basis of rice anthocyanin biosynthesis, we can manipulate this pathway to create ideal rice varieties with anthocyanins accumulated in specific organs as wished. As an edible part for humans, rice endosperm cannot synthesize anthocyanins, which greatly limits anthocyanin uptake for people who rely on rice as a staple food. One way is to consume whole-grain rice to maximize the health benefits of anthocyanins [26]. It requires that these black rice varieties should have good cooking quality and palatability. Another way is to create rice varieties with enriched anthocyanins in endosperm. It is delightful that through transferring eight anthocyanin-related genes controlled by distinct endosperm-specific promoters, the purple endosperm rice has been produced successfully [27]. However, this transgenic product contained two transcription factors from maize and six structural genes from coleus. Ideally, it is necessary to synthesize anthocyanins in endosperm by using rice endogenous genes instead of foreign genes. Nonetheless, when taking either approach, it is essential to understand how many rice anthocyanin-related genes are needed for accumulation of anthocyanins in endosperm or specific organs in natural rice germplasm.

In this study, we identified rice culm-specific pigmentation gene by map-based cloning. We further identified the WD40 protein of MBW members related to anthocyanin biosynthesis by homologous cloning. Through investigation of the regulatory relationship among the three members of MBW complex, we found that *S1* is the master key of the regulatory pathway. S1 activates the expression of *C1*, and C1 activates the expression of *WA1*. This hierarchical regulatory mechanism differs from that has been found in other plants. Based on in-depth analysis of regulatory network and natural variation of related genes, we preliminarily identified the minimum number of genes required for anthocyanin biosynthesis in rice germplasm. These findings will help us manipulate the molecular regulatory pathway of anthocyanin biosynthesis to create new rice varieties which meets specific health demands.

## 2. Results

### 2.1. S1 Determines Rice Culm Pigmentation

**Figure 1:**
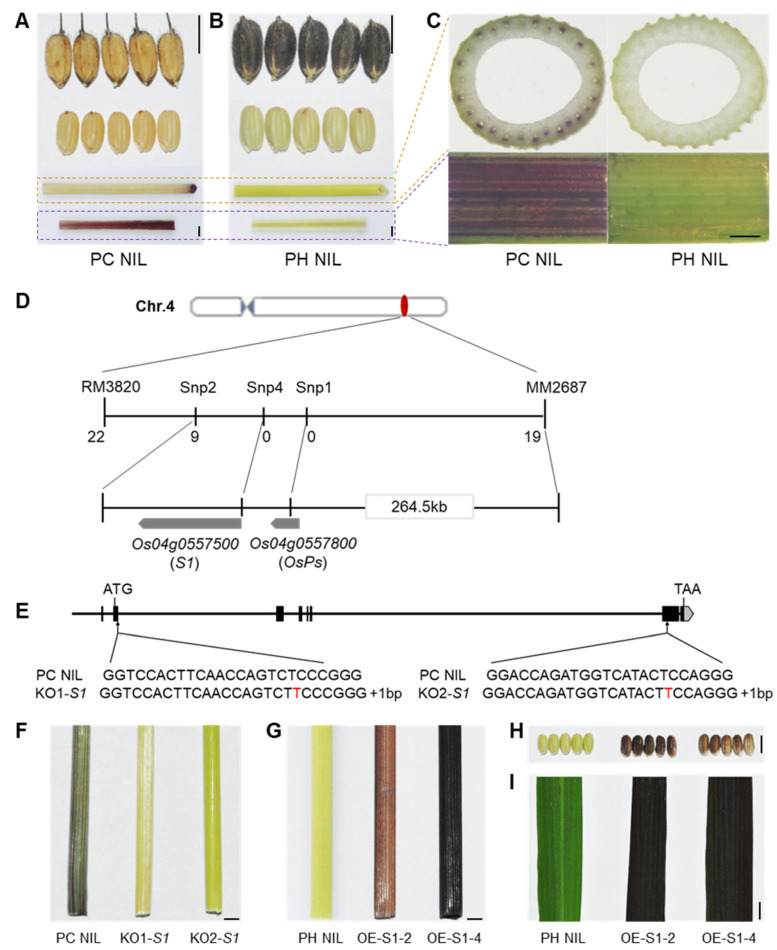
*S1* determines specific organ pigmentation. (**A**,**B**) Phenotypes of organ color in PC NIL (**A**) and PH NIL (**B**). From top to bottom: hull, pericarp, culm, sheath. (**C**) Cross-section of culm (top) and enlarged inner part of leaf sheath (bottom). (**D**) Map-based cloning of culm-specific pigmentation gene. (**E**) Knock-out of *S1* by CRISPR/Cas9 system. Two loss of function mutants were shown as KO1-*S1* and KO2-*S1* with a nucleotide ‘*T*’ inserted in the second or seventh exon, respectively. (**F**) The culm color of PC NIL and *S1* knockout lines at flowering stage. (**G**–**I**) The colors of culm (**G**), pericarp (**H**) and leaf blade (**I**) in *S1* overexpression transgenic lines (OE-S1-2 and OE-S1-4) with PH NIL background. The organ colors were shown at mature stage. Bar = 5 mm in A, B, F–I; Bar = 1 mm in C.

Anthocyanins can be accumulated in various organs of rice such as the hull, apiculus/awn, pericarp, stigma, culm, leaf, and leaf sheath (Figure S1). However, related genes regulating culm specific pigmentation has not been elucidated. In a previous study, we constructed two distinct near-isogenic lines (NILs) in a Nipponbare background with purple culm (PC) NIL and purple hull (PH) NIL, which separately accumulated anthocyanins in the culm and hull (Figure 1A,B). This indicates that the anthocyanin biosynthesis pathways of both NILs are unimpeded, but the main differences are which organs are pigmentated. PC NIL has a purple color in the culm and the inner part of the leaf sheath due to the accumulation of anthocyanins in its vascular bundles, whereas PH NIL shows a straw-yellow culm (Figure 1C).

By crossing PC NIL to PH NIL, we obtained F_1_ plants which exhibited a purple culm. F_2_ individuals segregated as purple culm (*n* = 211) and colorless culm (*n* = 62), and a clear 3:1 ratio (χ^2^_3:1_ = 0.25, *p* > 0.05) indicated that one dominant gene was responsible for the purple coloration in culm. Through linkage analysis, the candidate gene was mapped to an interval of 264.5 kb between markers MM2687 and Snp2 on chromosome 4 (Figure 1D). As predicted in RAP-DB, two cloned genes (*S1* and *OsPs*) are associated with anthocyanin biosynthesis located in this candidate region. *S1* has been known to regulate pericarp and hull color formation, whereas *OsPs* is responsible for stigma coloration. By comparing their DNA sequence between PC NIL and PH NIL, *S1* shows two SNPs in the promoter region, whereas *OsPs* shows two SNPs in the exon which caused amino acid institutions (Figure S2A,B). The candidate gene for culm pigmentation cannot be inferred from the DNA sequence.

We then knocked out the two genes in PC NIL by using the CRISPR/Cas9 system. Two different mutant lines of *S1* with 1 bp insertion in the first exon or the sixth exon showed a colorless phenotype in the culm (Figure 1E,F). However, the mutant with no function of *OsPs* still showed purple culm (Figure S2C). This demonstrates that *S1* is the causal gene for culm pigmentation. Overexpression of *S1* in PH NIL (OE-S1-2 and OE-S1-4) resulted in the accumulation of anthocyanins in culm, pericarp, and leaf tissues (Figure 1G–I). This implies that the expression level of *S1* in certain tissue is essential for anthocyanin pigmentation. Through qPCR analysis, we found the expression levels of structural genes for anthocyanin biosynthesis were significantly increased in *S1* overexpressing lines (Figure S3). This indicates that S1 promotes anthocyanin accumulation by activating the expression of structural genes.

### 2.2. Identification and Characterization of WD40-Encoding Gene for Anthocyanin Biosynthesis in Rice

**Figure 2:**
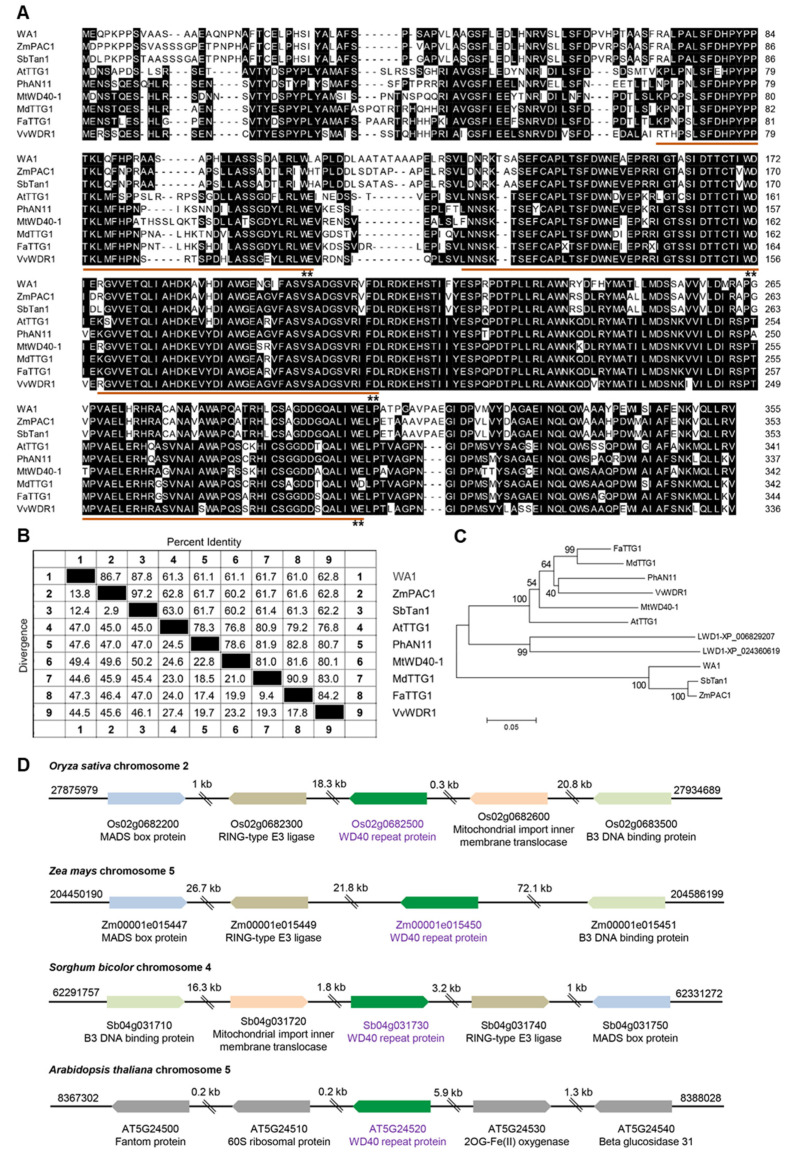
Conservation of anthocyanin-related WD40 proteins in plant species. (**A**) Protein sequence alignment of WA1 in rice and cloned WD40 proteins in other plants. The brown underscores indicate WD40 repeats and the stars marked the amino acids of W and D. (**B**) Matrix analysis of percent identity and divergence among WD40 proteins in plants. (**C**) Phylogenetic tree of anthocyanin-related WD40 proteins in plant species. ZmPAC1 (AAM76742), AtTTG1 (Q9XGN1), SbTan1 (AFN17366), PhAN11 (AAC18914), MtWD40-1 (ABW08112), MdTTG1 (ADI58760), FaTTG1 (JQ989287), VvWDR1 (ABF66625), LWD1-XP_006829207 (Amborella trichopoda), LWD1-XP_024360619 (Physcomitrium patens). (**D**) Regional synteny of anthocyanin-related WD40 gene in rice, maize, sorghum, and Arabidopsis.

The MBW complex has been elucidated to regulate anthocyanin biosynthesis in plants. However, as a member of MBW, WD40 proteins associated with anthocyanin biosynthesis in rice is largely unknown and their regulating mechanism needs to be further studied. We searched protein sequences for putative rice WD40 for anthocyanin biosynthesis in the National Center for Biotechnology Information (NCBI) by using BLASTp, by querying the characterized *Zea mays* ZmPAC1 [28]. Only one homolog (XP_015626852) with an identity of 87.9% was found in rice. We named its coding gene as *WA1* (WD40 for Anthocyanin biosynthesis, *Os02g0682500*). By comparing protein sequences of anthocyanin-related WD40 genes, which had been clarified in other plant species, we found that each homolog had four WD40 repeats and they were conserved in monocots and eudicots (Figure 2A). The similarities of WD40 protein sequences among species were all greater than 60%, and WA1 was grouped with ZmPAC1 and SbTan1 in a single clade (Figure 2B,C). Regional synteny analysis revealed that *WA1* neighboring genes are conserved in rice, maize, and sorghum, but are completely different from that in *Arabidopsis* (Figure 2D). This suggests that the biological function of WA1 and its homologs are conserved in monocots.

Through comparison of the DNA sequence of *WA1* between PH NIL and Nipponbare, no difference was found in the whole gene region including the promoter and the 3′-UTR. To identify natural variations of *WA1* in germplasm, we investigated the haplotype of it. There are mainly eight haplotypes for *WA1* (Figure S4). Hap1 is a *japonica*-specific haplotype, and Hap2/3 are *indica*-specific haplotypes. Hap4–Hap8 are specific in wild rice. It shows that *WA1* is obviously differentiated between *indica* and *japonica* subspecies. Only one nonsynonymous SNP in the first exon caused the 30th amino acid to change from Ile to Val. Moreover, several SNPs were also found in the promoter region. However, these nucleotide variations do not correlate with color change in rice germplasm. This suggest that *WA1* has no functional variation for rice color formation in the natural population.

**Figure 3:**
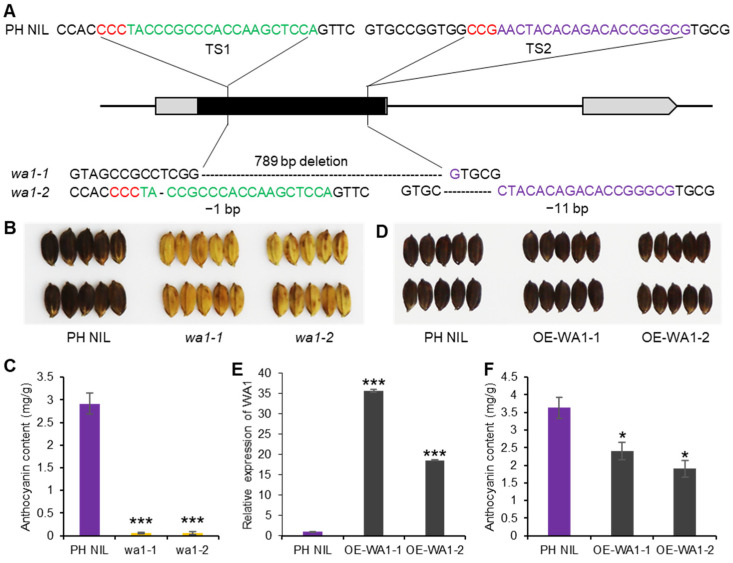
Functional validation of *WA1* for anthocyanin biosynthesis. (**A**) Knock-out of *WA1* by CRISPR/Cas9 system. Two loss-of-function mutants were shown as *wa1-1* and *wa1-2*. The red letters are PAM sequence, green and purple letters represent sgRNA target sites. (**B**) The hull color of PH NIL and *WA1* knockout lines. (**C**) Total anthocyanin content of PH NIL and *WA1* knockout lines. (**D**) The hull color of PH NIL and *WA1* overexpression lines (OE-WA1-1 and OE-WA1-2). (**E**) Expression level of *WA1* in PH NIL and *WA1* overexpression lines. (**F**) Total anthocyanin content of PH NIL and *WA1* overexpression lines. Data represent means ± s.e.m (*n* = 3 biological replicates). The asterisks indicate statistical significance by two-tailed Student’s *t*-tests (* *p* < 0.05, *** *p* < 0.001).

To verify the function of *WA1*, we knocked out *WA1* in PH NIL which had a purple hull. The *wa1-1* mutant has a 789-bp deletion in *WA1* exon, and *wa1-2* has 1-bp and 11-bp deletions in the different regions of the exon which caused a frame shift (Figure 3A). The hull color of the two mutants all changed from blackish purple to straw-yellow, resulting from the non-accumulation of anthocyanins in the hull (Figure 3B,C). This indicates that *WA1* is indispensable for anthocyanin biosynthesis in rice. In addition, we overexpressed *WA1* in PH NIL (OE-WA1-1 and OE-WA1-2) to see if it could promote anthocyanins accumulated in hull or other organs like *S1* (Figure 1G–I). We found that the hull color of *WA1* overexpressing lines could not be distinguished from PH NIL by the naked eye (Figure 3D,E). However, the total anthocyanin content was significantly decreased in the hulls of *WA1* overexpressing lines than that in PH NIL (Figure 3F). This indicates that excess WA1 proteins may repress the anthocyanin biosynthesis to some extent or activate potentially competing pathways. Apart from this, there are no anthocyanins accumulated in other organs of the overexpressing transgenic plants. This suggests that the biological function of *WA1* is different from that of *S1*.

### 2.3. WA1 Activates Anthocyanin Biosynthesis Pathway by Interacting with C1 and S1

**Figure 4:**
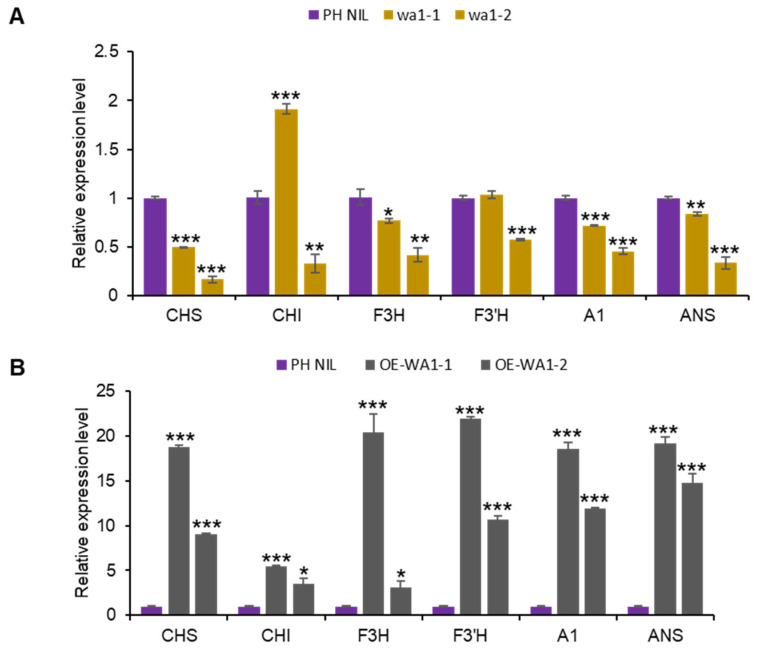
WA1 functions in the nucleus and regulates anthocyanin biosynthesis genes expression. (**A**,**B**) Expression of structural genes in *WA1* knockout lines (**A**) and *WA1* overexpression lines (**B**). Data represent means ± s.e.m (*n* = 3 biological replicates). The asterisks indicate statistical significance by two-tailed Student’s *t*-tests (* *p* < 0.05, ** *p* < 0.01, *** *p* < 0.001).

Through expression analysis, we found the expression of anthocyanin biosynthesis genes were significantly decreased in *WA1* knock out lines, but significantly increased in *WA1* overexpressing lines (Figure 4A,B). This demonstrates that *WA1* affects anthocyanin biosynthesis by mediating the expression of structural genes. Sub-cellular localization analysis revealed that WA1 is localized in the nucleus (Figure S5), which further indicates the function of WA1 in regulating gene expression through cooperation with other transcription factors.

**Figure 5:**
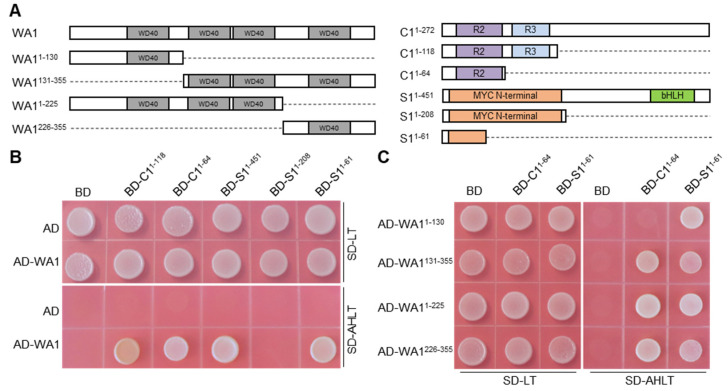
Y2H assay of WA1 with S1 and C1. (**A**) Schematic diagram of the WA1, C1, and S1 intact proteins and truncated proteins used for assay. The conserved domains of these proteins were shown as in the colored box. (**B**) WA1 interacted with R2 domain of C1 and N terminus of S1^1-61^. (**C**) WD repeat units interacts with R2 domain of C1 and N terminus of S1^1-61^. AD, pGADT7 empty vector; BD, pGBKT7 empty vector. SD-LT, SD Base medium +DO Supplement-Leu/-Trp; SD-AHLT, SD Base medium +DO Supplement-Ade/-His/-Leu/-Trp.

To test whether WA1 interacts with other MBW members to regulate structural genes expression, we performed yeast two hybrid analysis. The results showed that WA1 interacted with the R2 domain of C1 and the N terminus of S1^1-61^ (Figure 5A,B). It had been clarified that S1 interacted with C1 via binding to the R3 domain of C1 [15]. S1 and WA1 interacted with C1 in different domains, which provides a reasonable physical space for the formation of MBW complex. In addition, truncated WA1 proteins with one or three WD40 repeats do not affect its interaction with C1 and S1 (Figure 5C). Multi-binding sites enables WA1 to easily bind to its interacting proteins, which helps maintain the stability of the MBW complex. Therefore, we speculate that WA1 regulates the expression of anthocyanin biosynthesis genes by interacting with C1 and S1.

### 2.4. Hierarchical Regulation between MBW Members

**Figure 6:**
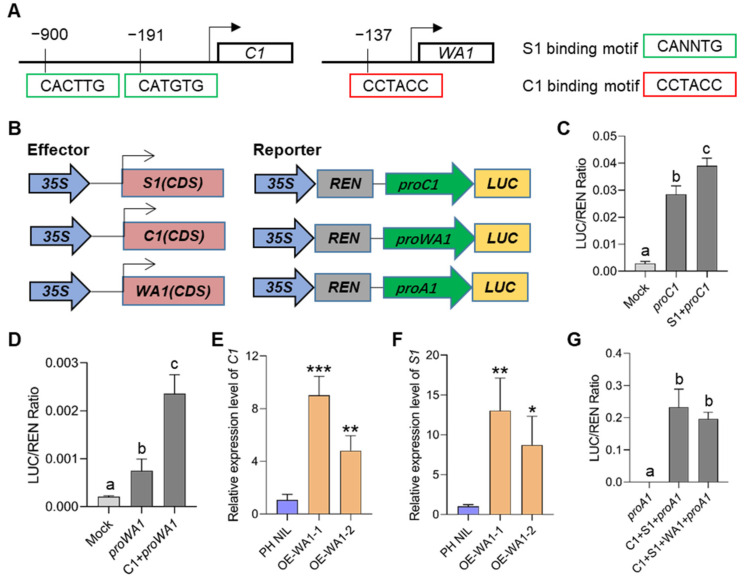
Hierarchical regulatory patterns among MBW members. (**A**) Schematic diagram showing S1 binding sites in the *C1* promoter, and C1 biding sites in the *WA1* promoter. (**B**–**D**) Transient expression assays of dual-luciferase by co-transfecting rice protoplasts with the vector shown in B. S1 can activate the expression of *C1* (**C**) and C1 can activate the expression of *WA1* (**D**). (**E**,**F**) Expression of *C1* (**E**) and *S1* (**F**) in *WA1* overexpression lines. (**G**) Transient expression assays of MBW members in activating *A1* which encodes a DFR (dihydroflavonol reductase). Error bars indicate the SD of three biological replicates. Different letters indicate statistically significant differences at *p* = 0.01 by one-way ANOVA test. The asterisks indicate statistical significance by two-tailed Student’s *t*-tests (* *p* < 0.05, ** *p* < 0.01, *** *p* < 0.001).

Coordinating the expression of MBW members is important for regulating the downstream biosynthesis genes under a feedback loop, which has been confirmed in other plant species. To demonstrate whether there is a transcriptional regulation between *C1*, *S1*, and *WA1*, we first predicted the candidate binding motifs in the promoter regions of these three genes. We identified two S1 binding sites in the *C1* promoter and one C1 binding site in *WA1* promoter (Figure 6A). Transient transcriptional activation analysis by using rice protoplasts showed that S1 could significantly activate the promoter activity of *C1*, and C1 could also significantly activate the promoter activity of *WA1* (Figure 6B–D). These suggest that there exists a hierarchical regulatory mechanism between MBW members of which S1 regulates *C1* and C1 regulates *WA1*. We also found that the expression of *C1* and *WA1* significantly increased in *S1* overexpressed transgenic lines in the Nipponbare background (Figure S6A–C). However, overexpression of *S1* in PH NIL did not significantly increase the expression of *C1* and *WA1*, but decreased the expression of *C1* (Figure S6D–F). These findings suggest that an unknown feedback regulation may also exist, which prevents the expression of *C1* and *WA1* from being continuously enhanced under the high expression of *S1*, so that the number of MBW complexes and anthocyanins content can be controlled at a certain level. In short, we suggest that *S1* plays a central role in the cascade regulation of MBW members.

Does WA1 affect the expression of *C1* and *S1*? To answer this question, we analyzed the expression of *C1* and *S1* in *WA1* overexpressing lines. Expression of both genes was found to be more significantly increased in overexpressing transgenic lines than that in the PH NIL (Figure 6E,F). This may be caused by the increased number of MBW complex which in turn activated the expression of *C1* and *S1*. However, in the process of activating downstream structural genes, when the amount of C1 and S1 are excessive, overexpression of *WA1* cannot further enhance the activation of downstream genes (Figure 6G), which further indicates the existence of feedback inhibition in the transcriptional regulation of anthocyanin biosynthesis. Therefore, we speculate that *WA1* is more likely to be involved in regulating rice anthocyanin biosynthesis by stabilizing MBW transcription complex.

### 2.5. De-Novo Design Colored Rice

**Figure 7:**
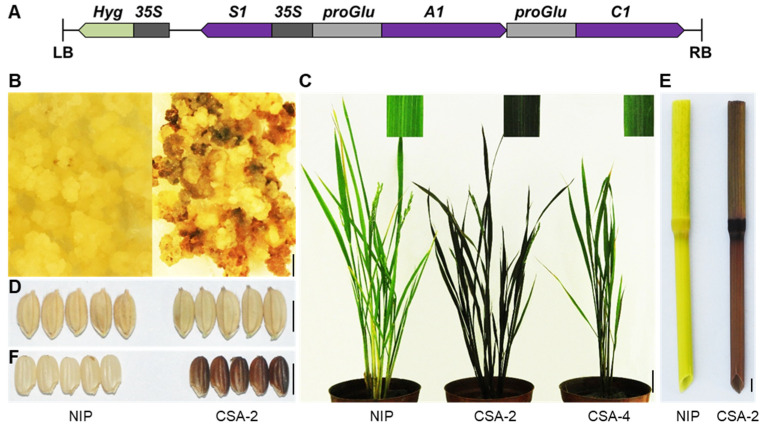
De-novo design colored rice. (**A**) Schematic illustration of recombinant construct *CSA*. *S1* is promoted by CaMV35S, *A1* and *C1* are driven by the promoter of *OsGluA2*, which is highly expressed in the endosperm. (**B**–**F**) The purple anthocyanins accumulated in *CSA* transgenic lines such as callus (**B**), leaf blade (**C**), apiculus (**D**), culm (**E**), and pericarp (**F**). NIP, Nipponbare. Bar = 5 mm in B, D, E and F. Bar = 5 cm in C.

The number of genes needed in anthocyanin accumulation in specific organs of rice varieties through transgenic approaches is an interesting topic to discuss. It has been clarified that anthocyanins cannot be synthesized in most rice cultivar owing to the loss-function of *C1* and *A1* [15]. As *S1* is a causal gene for pigmentation in most organs. Therefore, we consider that the functional *C1* and *A1* combining *S1* are a minimal set of genes required for anthocyanin biosynthesis in rice. To test it, we used Nipponbare as the transformation receptor material. The *C1* and *A1* are loss of function in Nipponbare, and no anthocyanins are accumulated in it. We constructed a vector that simultaneously overexpressed *S1*, and specifically expressed *C1* and *A1* driven by the promoter of *OsGluA2* which encodes a glutelin type-A2 precursor (Figure 7A). After being infected by *Agrobacterium*, the calli began to accumulate anthocyanins, demonstrating that this construct could effectively promote anthocyanin biosynthesis in Nipponbare (Figure 7B). The positive transgenic lines showed a dark purple color in leaf, culm, and apiculus (Figure 7C–E). More importantly, the pericarp color also changed to purple (Figure 7F), which is promising for breeding black rice varieties. Through this preliminary trial, we demonstrated the manipulation of the gene regulatory network of anthocyanin biosynthesis in rice, which will provide a concept for further analysis of molecular mechanism of rice organ pigmentation, and which will eventually help to achieve precise regulation of anthocyanin biosynthesis in target organs.

## 3. Discussion

Hierarchical regulation of transcription factors is universal in cells, which is crucial for efficiently regulating gene network in a coordinated manner. In this study, we investigated the genetic and molecular mechanisms of the MBW members in regulating anthocyanin biosynthesis in rice, and proved that *S1* is the superior of this regulatory network. S1 regulates the expression of *C1*, and then C1 regulates the expression of *WA1*, forming a cascade regulation model between MBW members. This regulation strategy enables a coordination between MBW members, which can efficiently promote the formation of MBW complex. The WD40 proteins may function as scaffolding proteins that connect bHLH and MYB [29,30]. Then the anthocyanin biosynthesis genes are activated by C1 and S1 directly binding to their promoter, as almost all structural genes have C1 and S1 binding sites in the promoters (Figure S7). Meanwhile, the highly activated downstream genes will also affect the expression level of upstream genes, so that the whole regulatory pathway can be precisely controlled in a feedback loop. In plants, the MBW member genes are conserved in protein sequences, but the hierarchical regulation mechanisms among the three kinds of genes are diverse. In rice, bHLH is considered as the core regulator for manipulating the anthocyanin biosynthesis. However, in Arabidopsis, MYB is thought to play a central role for anthocyanin biosynthesis [24,25]. In petunias, ectopic expression of *AN2* (MYB) induces *AN1* (bHLH) expression in leaves, whereas in anthers, *AN1* expression depends on AN4 (MYB), a paralog of AN2 [31]. *AN11* encodes a WD40 repeat protein that is expressed independently from *AN1* and *AN2* throughout plant development. Overexpression of *AN2* in *an11^-^* petals restored the activity of structural gene in transient assays, indicating that *AN11* acts upstream of *AN2* [32]. Taken together, this indicates that AN11 should regulate *AN2*, and AN2/4 should regulate *AN1*. In maize, the genetics of the anthocyanin biosynthesis pathway has been widely studied. *B* (bHLH), *C1* (MYB), and *PAC1* (WD40) are all required for activating the expression of anthocyanin biosynthesis genes [28,33]. The expression of *B* and *C1* are similar in *pac1-ref* and wild-type individuals, suggesting that *PAC1* does not influence the expression of either of these genes. It also demonstrates that *PAC1* expression does not require *B* or the exclusive action of *C1* or *pl1* [34]. There is no evidence that any of these three regulators regulate each other. Comparing eudicots and monocots, we found that regulatory genes in anthocyanin biosynthesis pathways are relatively conserved in different plant species, but their regulatory mechanism are indeed diverse. These opposite regulatory strategies between bHLH and MYB transcription factors implying the functional specialization of these genes in different plant species, which can ultimately result in the differences in pigmentation patterns among species. The biological significance of these different regulatory strategies remains an open question.

Multiple studies have revealed that *bHLH* genes are essential for organ-specific pigmentation in rice. An insertion in the promoter of *S1* (also known as *Kala4*) caused ectopic expression of it and finally accumulated anthocyanins in the pericarp [19]. Hull-specific pigmentation is also determined by *S1* [15]. What interested us is that *OsPs* and *OsPa*, both encoding bHLH transcription factors, are specific for stigma and apiculus pigmentation, respectively [20]. In addition, by developing near-isogenic lines for the purple leaf, we mapped the candidate gene region which also contained *S1* (data not shown). In this study, we clarified that *S1* also controlled culm pigmentation. Through overexpression of *S1* in PH NIL, we also found that the colors of pericarp, hull, culm, and leaf changed to purple. The same results have been found in PC NIL along with an overexpression of *S1* [15]. These indicate that if *S1* is highly expressed in an organ, it can activate the entire metabolic pathway to synthesize anthocyanins. Nevertheless, overexpression of *WA1* or *C1* did not cause accumulation of anthocyanins in these organs simultaneously. This suggests that *S1*, but not *C1* or *WA1*, is essential for specific anthocyanin pigmentation in pericarp, hull, culm, and leaf. However, except for pericarp pigmentation, the functional variations of *S1* for pigmentation of other three organs are yet to be discovered. The molecular regulation mechanism for organ-specific pigmentation still needs to be studied further.

It is an efficient short cut to develop rice varieties with pigmentation in specific organs by transgenic approach. The most successful example is the creation of purple endosperm rice [27]. However, it required transferring eight anthocyanin-related genes into rice at a time, which is undoubtedly difficult. In fact, according to our genetic studies on anthocyanin biosynthesis in rice, most of the structural genes are functional except *A1* (DFR). Therefore, we speculate that it may be feasible to synthesize anthocyanins in endosperm of most rice accessions by transferring *C1*, *S1*, and *A1* genes altogether. This should be easier for transformation and, more importantly, all the transformed genes are cloned from rice without introducing exogenous genes. However, in this study, the synthesis and accumulation of anthocyanins in the endosperm were not successfully achieved. It may be due to the lower activation efficiency of the endosperm-specific promoter used in this study or the inability of *C1* and *S1* to effectively activate the expression of structural genes in the endosperm. Nevertheless, we have preliminarily confirmed that a minimum set of genes required for anthocyanin biosynthesis in rice organs, which provides valuable guidance for accumulation of these compounds in rice endosperm by transforming endogenous genes. In future studies, we will focus on the utilization of other highly expressed promoters in endosperm to drive the expression of anthocyanin-related genes.

## 4. Material and Methods

### 4.1. Plant Materials and Growth Conditions

PC NIL (also known as PA NIL in previous studies), a NIL of BC_4_F_8_ with purple culms and awn, was obtained from the crosses and backcrosses between Nipponbare and a temperate *japonica* with purple culms and awns [15].

PH NIL is a NIL of BC_2_F_6_ with a purple hull in the Nipponbare background. The donor parent was a temperate *japonica* variety with purple hulls [15].

All rice plants were grown under natural conditions in paddy fields located in Beijing, China.

### 4.2. Plasmid Construction for Rice Transformation

The vector for overexpression of *S1* was constructed by amplifying the *S1* (*Os04g0557500*) coding DNA sequence (CDS) from cDNA of PH NIL. The PCR products were digested with *BamH* I and *Spe* I, followed by cloning into the binary vector pMDC32 [35]. For overexpression of *WA1*, the fragments containing CDS of *WA1* (*Os02g0682500)* was amplified from cDNA of PH NIL and cloned into the modified pCAMBIA1307. To construct the *S1* and *OsPs* knock-out mutants, the target site primers were synthesized and integrated into sgRNA-Cas9 [36]. For generation of *WA1* knock-out mutant by CRISPR/Cas9, two target site fragments were cloned into the binary vector pHUE411. For constructing the *CSA* co-expression vector, the promoter of *OsGluA2* (*Os10g0400200*) was used for driving the transcription of *C1* and *A1*, and *S1* was driven by the CaMV35S promoter. The three fused fragments were cloned into a pCAMBIA1300 vector. The recombinant plasmids were transformed into rice calli by *Agrobacterium tumefaciens* [37]. The sequences of all plasmids were provided in the Table S1.

### 4.3. Gene Expression Analysis

The total RNA was extracted from leaf or culm tissues by using RN03-RNApure Kit (Aidlab Biotech, Beijing, China). First-strand cDNA was synthesized by using the PC54-TRUEscript RT kit (+gDNA Eraser) (Aidlab Biotech, Beijing, China). qRT-PCR was performed by using TB Green^®^ Premix Ex Taq™ (Tli RNaseH Plus) (Takara, San Jose, CA, USA). The ubiquitin gene *Os03g0234200* was used as an internal reference. The sequences of primers used for PCR were listed in Table S2.

### 4.4. Yeast Two-Hybrid Assay

Full-length CDS of *WA1* and its truncated CDS were amplified from the cDNA of PH NIL and subcloned into the pGADT7 vector (Takara, San Jose, CA, USA) between *EcoR* I and *BamH* I sites. Full-length or truncated CDS of *C1* and *S1* were fused into the pGBKT7 vector between the *EcoR* I and *BamH* I sites. Transformed yeast cells were grown on SD-Trp/-Leu (-LT) or SD-Trp/-Leu/- His/-Ade(-AHLT) media. Experimental procedures were performed according to the manufacturer’s user manual (Clontech). Yeast strain AH109 was used in this assay.

### 4.5. Transient Activation Assay

The full-length CDS of *S1* and *C1* were amplified from the cDNA of PH NIL and cloned into the vector pGreenII 62-SK to generate effectors. To construct the reporter, the promoters of *C1* (1.3 kb upstream of the ATG) and *WA1* (1.2 kb upstream of the ATG) from PH NIL were amplified and cloned into the vector pGreenII 0800-LUC [38]. Transactivation analysis was performed in the protoplasts extracted from 3-week-old leaf sheath of Nipponbare. Transfected protoplasts were incubated in the dark for 16 hours at 28℃, and then cells were lysed, and the luciferase activity was detected by using the Dual-Luciferase Reporter Assay System (Promega, E1960) (Madison, WI, USA). The Renilla luciferase (REN) gene was used as an internal control.

### 4.6. Sub-Cellular Localization

The CDS of *WA1* without a stop codon was amplified from the cDNA of PH NIL, and inserted into the pCAMBIA super1300-GFP driven by constitutive promoters to generate a fusion protein with GFP fused to the C terminus of WA1. The generated construct was transformed into protoplasts extracted from 3-week-old leaf sheath of Nipponbare. Fluorescences of protoplasts were examined under a confocal laser-scanning microscope (ZEISS LSM900). The following excitation (Ex) and emission (Em) wavelengths were used for detection: GFP (Ex = 488, Em = 500–550).

### 4.7. Examination of Total Anthocyanins

The hull samples were collected from the matured grains. An equal amount of hull sample was collected from 10 individual plants and mixed. The samples were ground in liquid nitrogen and approximately 0.5 g powder was extracted by 5 mL methanol supplemented with 0.1% HCl at 4 °C for 24 h. After centrifugation for 10 min at 10,000× *g*, the supernatant was filtered through a 0.22-μm syringe filter. Four milliliters of sodium acetate buffer (0.4 mol/L, pH 4.5) or potassium chloride buffer (0.25 mol/L, pH 1.0) were added in 1 mL of supernatants. The absorbance of the anthocyanins was measured at 520 and 700 nm by using a Multiskan Spectrum (Thermo Scientific Multiskan GO 1510, Vantaa, Finland). The total content of anthocyanins per sample fresh weight was calculated according to the following formula: anthocyanin content (mg/g fw) = A/εL*10^3^*MW*D. A = (A_520 nm, pH1.0-700 nm, pH1.0_) − (A_520 nm, pH4.5_ − A_700 nm, pH4.5_). ε = 29,020, representing molar absorption coefficient of cyanidin 3-glucoside. Here, L is the optical path length. MW stands for molecular weight (cyanidin 3-glucoside, 484.84 g/moL). D is dilution factor [39]. Each sample was detected and assayed in three replications.

### 4.8. Callus Induction

Dehusked seeds were sterilized by 70% ethanol for 2 min, followed by 15% sodium hypochlorite solution for 20 min with shaking. The seeds were rinsed several times with sterile water on an ultraclean workbench. Dried seeds were cultured in NB medium (NB medium containing N6 macronutrient, B5 micronutrient components, organic, FeNaEDTA, moy-inositol, supplemented with 2 mg/L of 2,4-D and 30 g/L of sucrose) for callus induction and subculture. The pH of the medium was adjusted to 5.8, and 3 g/L of phytagel was added before autoclaving at 121 °C for 20 min. The seeds were incubated in an induction medium for 10 days at 28 °C in the dark. The induced calli were detached and transferred to a new NB medium growing in the dark at 28 °C for 3 weeks. Then, the calli were used for transformation mediated by *Agrobacterium tumefaciens*.

## Data Availability

Data supporting the findings of this work are available within the paper and its Supplementary Information files. The datasets and genetic materials supporting the findings of current study are available from the corresponding authors upon request.

## Supplementary Materials

The following supporting information can be downloaded at: https://www.mdpi.com/article/10.3390/ijms23158203/s1.
