# Supplementary material for: Uncovering Hierarchical Regulation among MYB-bHLH-WD40 Proteins and Manipulating Anthocyanin Pigmentation in Rice
Source: Int J Mol Sci. 2022 Jul 26;23(15):8203. doi: 10.3390/ijms23158203 (PMC9332703; doi:10.3390/ijms23158203)
Supplement: Supplementary file 1 [file ijms-23-08203-s001.zip › ijms-1788798-supplementary/ijms-1788798-supplementary.pdf]

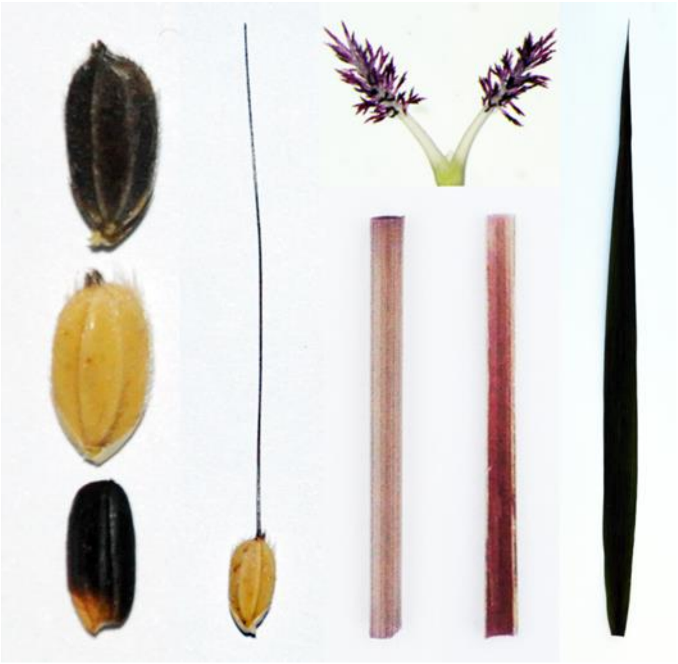

Figure S1. Anthocyanins pigmented organs in rice. From left to right: hull, apiculus, pericarp, awn, pistil, culm, leaf sheath, leaf blade.

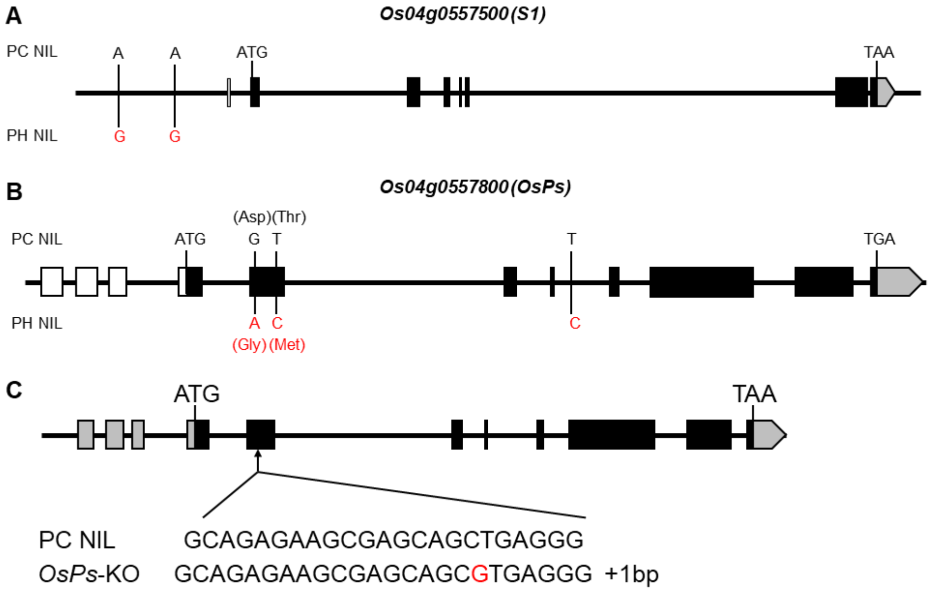

Figure S2. Schematic diagrams of gene structure. *S1* (**A**) and *OsPs* (**B**) show differences between PC NIL and PH NIL. (**C**) Knockout of *OsPs* by CRISPR/Cas9 system. A 'G' insertion in the second exon of the gene caused frame shift.

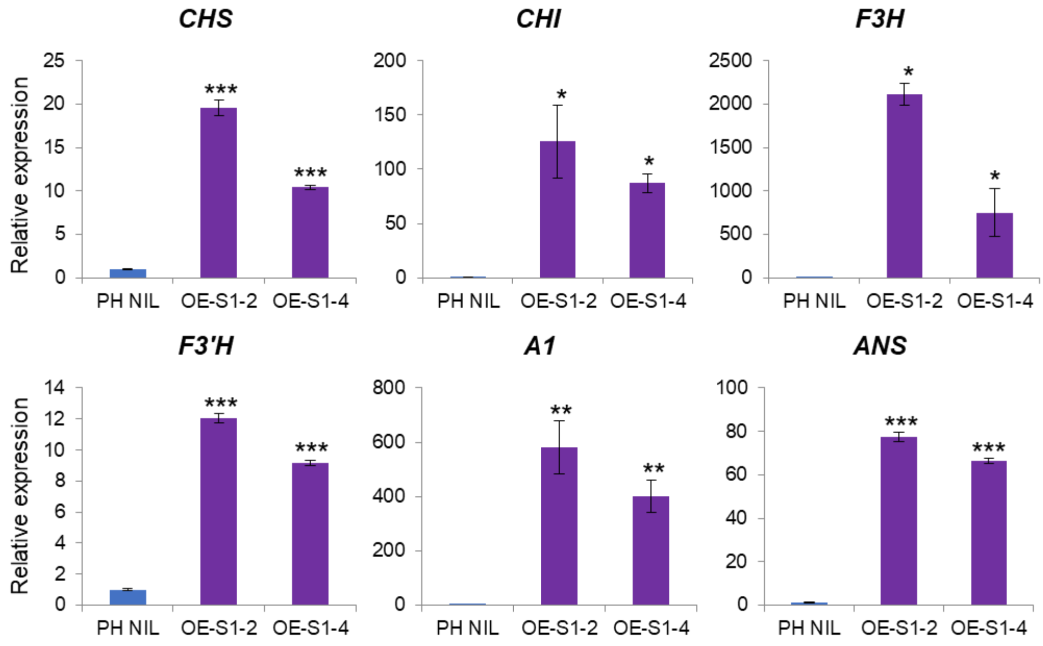

Figure S3. Expression of biosynthesis genes in *S1* overexpressed transgenic lines (OE-S1-2 and OE-S1-4). Data represent means ± s.e.m (n=3 biological replicates). The asterisks indicate statistical significance by two-tailed Student's *t*-tests (*P<0.05, **P<0.01, ***P<0.001).

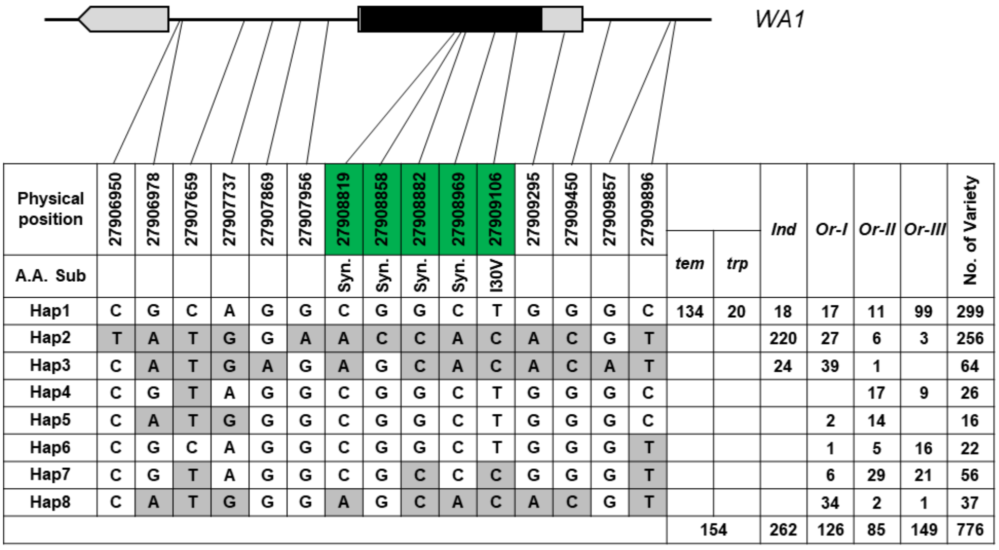

Physical position	27906950	27906978	27907659	27907737	27907869	27907956	27908819	27908858	27908882	27908969	27909106	27909295	27909450	27909857	27909896			*Ind*	*Or-I*	*Or-II*	*Or-III*	No. of Variety
A.A. Sub							Syn.	Syn.	Syn.	Syn.	I30V					*tem*	*trp*					
Hap1	C	G	C	A	G	G	C	G	G	C	T	G	G	G	C	134	20	18	17	11	99	299
Hap2	T	A	T	G	G	A	A	C	C	A	C	A	C	G	T			220	27	6	3	256
Hap3	C	A	T	G	A	G	A	G	C	A	C	A	C	A	T			24	39	1		64
Hap4	C	G	T	A	G	G	C	G	G	C	T	G	G	G	C					17	9	26
Hap5	C	A	T	G	G	G	C	G	G	C	T	G	G	G	C				2	14		16
Hap6	C	G	C	A	G	G	C	G	G	C	T	G	G	G	T				1	5	16	22
Hap7	C	G	T	A	G	G	C	G	C	C	C	G	G	G	T				6	29	21	56
Hap8	C	A	T	G	G	G	A	G	C	A	C	A	C	G	T				34	2	1	37
																154		262	126	85	149	776

Figure S4. Haplotype of *WA1*. Syn., synonymous mutation.

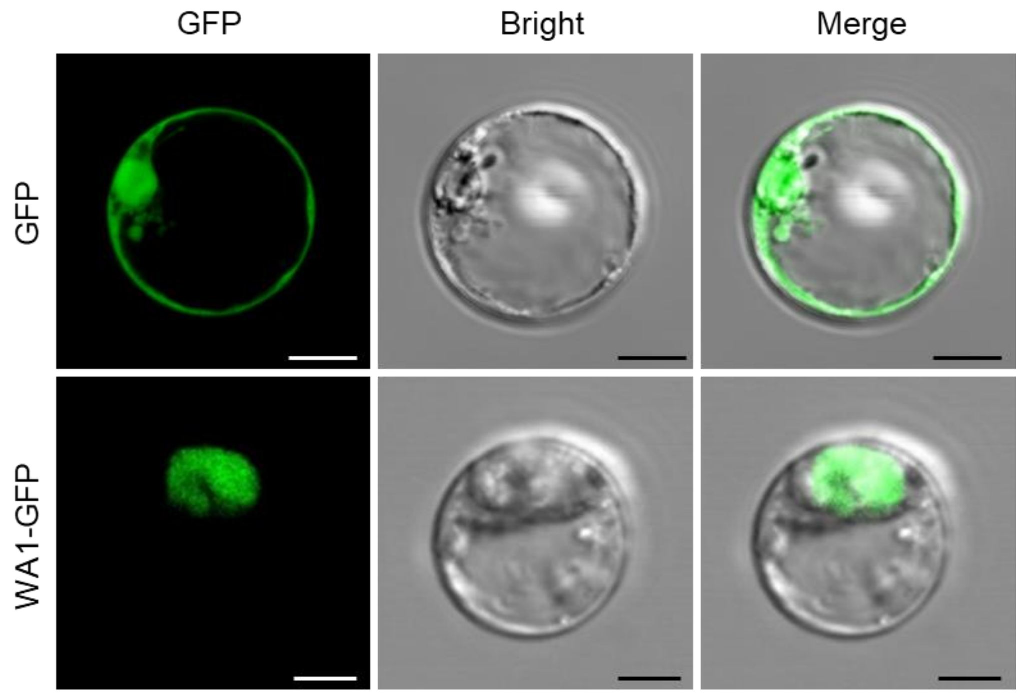

Figure S5. Sub-cellular localization of WA1 in rice protoplast. Bar=5 um.

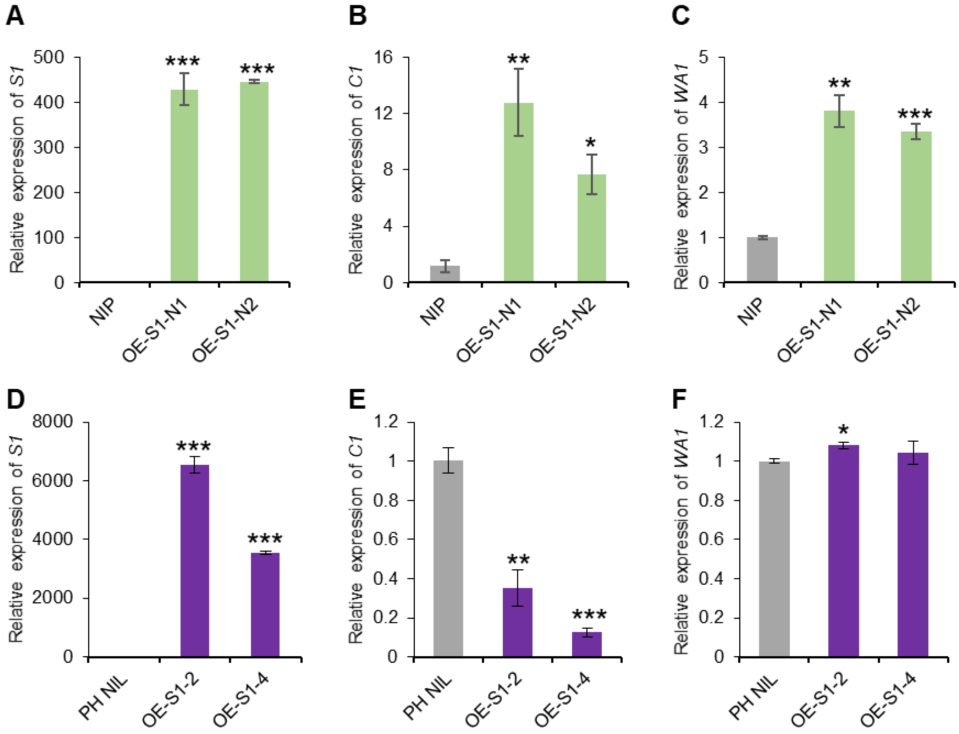

Figure S6. Expression of *S1*, *C1* and *WA1* in *S1* overexpressed transgenic lines. (**A-C**) *S1* overexpressed in Nipponbare. (**D-F**) *S1* overexpressed in PH NIL. Data represent means ± s.e.m (n=3 biological replicates). The asterisks indicate statistical significance by two-tailed Student's *t*-tests (*P<0.05, **P<0.01, ***P<0.001).

Promoters	C1 binding motif	S1 binding motif (CANNTG)
proCHS (Os11g0530600)	CCAACC(-171)	CACGTG(-262;-300)
proCHI (Os03g0819600)	CCAACC(-948)	CACTTG(-900)
proF3H (Os04g0662600)	CCTACC(-339)	CAGGTG(-692)
proF3'H (Os10g0320100)	CCTACC(-126)	CAGGTG(-228)
proA1 (Os01g0633500)	CCTACC(-470,-2017)	CACGTG(-2031) /CAGGTG(-484)
proANS (Os01g0372500)	CCTACC(-568)	CACGTG(-212)

Figure S7. Predicted binding sites of C1 and S1 in the promoter of structural genes.
